# Solubility enhancement of berberine–baicalin complex by the constituents of Gardenia Fruit

**DOI:** 10.1007/s11418-020-01446-1

**Published:** 2020-08-28

**Authors:** Kazuki Okoshi, Yoshinori Uekusa, Yuji Narukawa, Fumiyuki Kiuchi

**Affiliations:** Faculty of Pharmacy, Keio University, 1-5-30 Shibakoen, Minato-ku, Tokyo, 105-8512 Japan

**Keywords:** Solubility-enhancement, *Gardenia jasminoides*, Crocin, Orengedokuto, Berberine–baicalin complex

## Abstract

A Kampo prescription usually consists of several crude drugs and contains many kinds of compounds. Physicochemical interactions between the compounds may occur in the process of decoction, by which Kampo prescriptions are usually prepared for ingestion, and the interactions may change the extraction yields of the constituents. Berberine and baicalin have been reported to form precipitates. Orengedokuto, which consist of Coptis Rhizome, Gardenia Fruit, Phellodendron Bark and Scutellaria Root, has been a representative Kampo prescription used to treat inflammatory diseases. In our previous papers, we revealed that the precipitates formed in the decoction of orengedokuto without Gardenia Fruit mainly consists of berberine–baicalin complex and that Gardenia Fruit reduced the amount of the precipitates in orengedokuto decoction. In this report, through solubility-enhancement assay based on HPLC, we identified crocins as the constituents of Gardenia Fruits, which enhanced the solubility of berberine–baicalin complex. All-*trans* crocin-1 (**1**) and 13-*cis* crocin-1 (**5**) showed high activities among the isolated crocins, and the number of glucosyl groups in the molecule seemed correlated with the activity. As berberine and baicalin were reported as the anti-inflammatory constituents of Coptis Rhizome and Phellodendron Bark, and Scutellaria Root, respectively, Gardenia Fruit contributes anti-inflammatory activity of orengedokuto by increasing solubilities of anti-inflammatory constituents of the other component crude drugs in the prescription. Our result will add a scientific basis to the understanding of the effectiveness of orengedokuto as a whole.

## Introduction

A Kampo prescription usually consists of several crude drugs and contains many kinds of compounds. Physicochemical interactions between the compounds may occur in the process of decoction, by which Kampo prescriptions are usually prepared for ingestion, and the interactions may change the extraction yields of the constituents [1, 2]. Precipitate formation is one of the interactions causing such changes [3]. Interactions of berberine with baicalin [4], glycyrrhizin [5], tannins [3], and wogonoside [6] are typical examples of such precipitate formation out from a decoction.

Orengedokuto (黄連解毒湯, huanglianjiedutang), consisting of Coptis Rhizome, Gardenia Fruit, Phellodendron Bark and Scutellaria Root, contains berberine, a characteristic component of Coptis Rhizome and Phellodendron Bark, and baicalin, the major constituent of Scutellaria Root. Recent study showed that the yellow precipitates formed in decoction of orengedokuto were mainly composed of berberine and baicalin [4], and our group revealed that the constituents of Gardenia Fruit reduced the amount of berberine–baicalin precipitates in orengedokuto decoction [7]. Gardenia Fruit, the fruit of *Gardenia jasminoides* Ellis (Rubiaceae), is a crude drug used in Asian traditional medicines. Anti-hypertension [8], antioxidant [9], anxiolytic [10], choleretic [11], and neuroprotective [12] activities have been reported for Gardenia Fruit. Representative constituents of Gardenia Fruit are iridoid glycosides (geniposide, genipin gentiobioside, etc.) [13], crocins [14], monoterpenoids (jasminosides and sacranosides) [15], and quinic acid derivatives [16]. However, there has been no report on the compound responsible for the solubility-enhancement of the berberine–baicalin complex. In this paper, we describe the identification of the compounds in Gardenia Fruit involved in solubility-enhancement of berberine–baicalin precipitates.

## Materials and methods

### Materials and apparatus

**Fig. 1:**
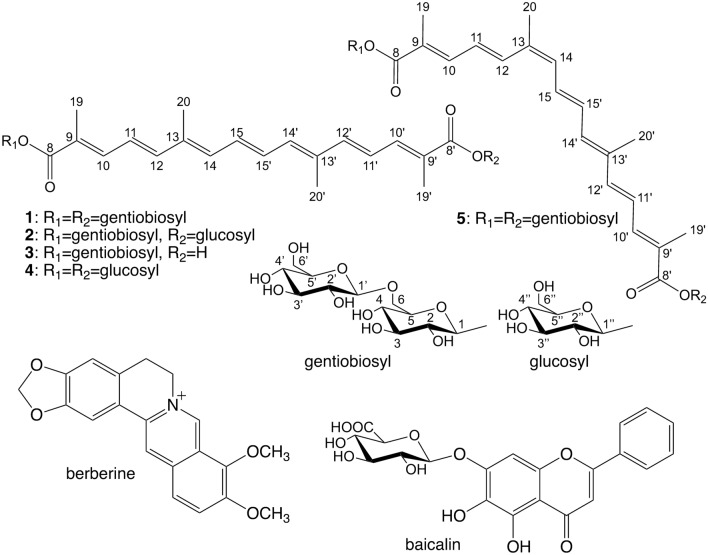
Structures of all-*trans* crocin-1 (**1**), all-*trans* crocin-2 (**2**), all-*trans* crocin-3 (**3**), all-*trans* crocin-4 (**4**), and 13-*cis* crocin-1 (**5**)

**Fig. 2:**
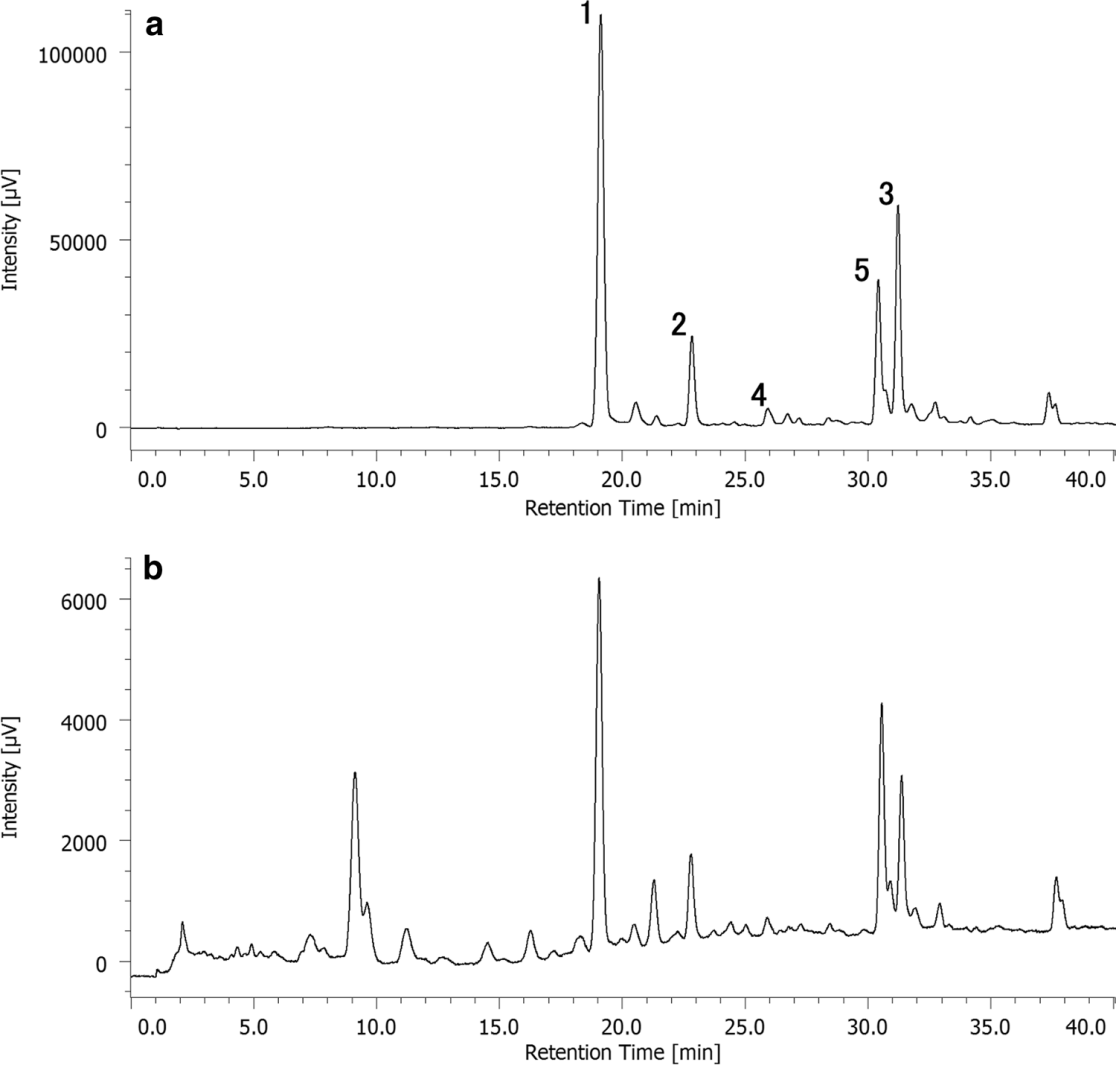
HPLC chromatograms of the commercially available crocin (Gardenia Fruits Extract) detected at 440 nm (**a**) and at 275 nm (**b**). The ratio of **1**–**5** was estimated to be about 21:4:9:1:6 from the peak areas in the chromatogram detected at 440 nm

Gardenia Fruit (Japanese Pharmacopoeia grade) was purchased from Tochimoto Tenkaido Co., Ltd. (Osaka, Japan, lot no. 004613004) and Uchida Wakanyaku Ltd. (Tokyo, Japan, lot no. c310250). Baicalin was purchased from FUJIFILM Wako Pure Chemical Corp. (Osaka, Japan). Berberine hydrochloride was purchased from Nacalai Tesque, Inc. (Tokyo, Japan). Crocin (Gardenia Fruits Extract) was purchased from Tokyo Chemical Industry Co., Ltd., (Tokyo, Japan, Lot. SSLBJ-MB) and used as a positive control. This material contained compounds **1**–**5** (Fig. 1), and their molar ratio was about **1**:**2**:**3**:**4**:**5**=21:4:9:1:6 from the peak areas of its HPLC chromatogram detected at 440 nm (Fig. 2). Ultrapure water prepared by an AQUARIUS^®^ (Advantec Co., Ltd., Tokyo, Japan) was used for the experiments. For HPLC analysis, LC/MS grade water (FUJIFILM Wako Pure Chemical Corp.) and HPLC grade methanol (Kanto Chemical Co., Inc., Tokyo, Japan) were used. TLC analyses were performed using TLC Silica gel 60 F254 or TLC Silica gel 60 RP-18 F254s (Merck Ltd., Tokyo, Japan). Mass spectra were acquired in electrospray ionization (ESI) with a time-of-flight (TOF) mass spectrometer operated in positive and negative ion modes (AccuTOF LC-plus JMS-T100LP, JEOL Ltd., Tokyo, Japan). ^1^H-NMR spectra were recorded on Varian Unity Inova 500 (Agilent Technologies Japan, Ltd., Tokyo, Japan) spectrometer operating at 500.20 MHz ^1^H resonance frequencies or Avance III HD (Bruker Japan K.K., Kanagawa, Japan) spectrometer operating at 500.17 MHz ^1^H resonance frequencies. Tetramethylsilane (TMS) was used as an internal standard. Preparative HPLC was performed on an LC-9201R system (Japan Analytical Industry Co., Ltd., Tokyo, Japan) equipped with an Inertsil PREP-ODS column (10 µm, 20 mm × 250 mm) and an Inertsil PREP-ODS guard column (10 µm, 20 mm × 50 mm, GL Sciences Inc., Tokyo, Japan). Chromatograms were monitored with a UV–VIS DETECTOR UV-3740 and recorded using JDS-200 (Japan Analytical Industry Co., Ltd.). For the HPLC based solubility assay, an HPLC system consisting of PU-2089 Plus pump, AS-2055 Plus autosampler, CO-2065 Plus column oven, UV-2070 Plus detector, and Chrom NAV data acquisition system (JASCO Corporation, Tokyo, Japan) was used. The analysis was performed using an Inertsil ODS-3 column (5 µm, 4.6 mm × 150 mm, GL Sciences Inc.) at 40ºC with a flow rate of 1.0 mL/min, and the eluate was monitored at UV 275 nm. Ultrapure water containing 0.1% formic acid (solvent A) and methanol containing 0.1% formic acid (solvent B) were used as mobile phase, and the gradient program was as follows: 0–10 min, 15–31% solvent B; 10–20 min, 31–80% solvent B; 20–25 min, 80% solvent B. Retention times of berberine and baicalin under this condition were 13.6 min and 20.5 min, respectively. For the analysis of commercially available crocin, the eluate was monitored at UV 275 nm and 440 nm, and the following gradient program was used: 0–10 min, 40% solvent B; 10–40 min, 40–80% solvent B.

### Extraction and isolation

Gardenia Fruit (50.1 g) was extracted with 450 mL of hot water under reflux for 1 h, and the extract was filtered. The residue was further extracted in the same way, and the combined extracts were lyophilized to give water extract (10.2 g). The water extract was applied to a Diaion HP-20 (Nippon Rensui Co., Tokyo, Japan) column (4 cm × 23 cm), and eluted with water–methanol (0%, 20%, 50%, and 100% methanol) and acetone to give 5 fractions (DW, 3.5 g; DM-1, 1.9 g; DM-2, 2.3 g; DM-3, 1.1 g; DA, 0.1 g, respectively).

For the isolation of crocins, Gardenia Fruit (496 g) was extracted with 1.5 L of 40% aqueous ethanol at room temperature for 24 h, and the extract was filtered. The residue was further extracted 3 times with 1 L of 40% ethanol for 24 h. The combined extracts were concentrated under reduced pressure and partitioned between water and ethyl acetate (1:1). After removal of ethyl acetate by partial concentration, the water layer was applied to a Diaion HP-20 column (6 cm × 21 cm) and eluted with water–methanol (0%, 20%, 50%, and 100% methanol) to give 4 fractions (C1, 67.7 g; C2, 21.0 g; C3, 21.8 g; C4, 12.9 g, respectively). Fraction C4 (2.36 g) was repeatedly separated by preparative HPLC with 60% methanol to give all-*trans* crocin-1 (**1**, 7.8 mg), all-*trans* crocin-2 (**2**, 4.7 mg), all-*trans* crocin-3 (**3**, 3.5 mg), and all-*trans* crocin-4 (**4**, 1.4 mg).

To isolate crocins more effectively, commercially available crocin (Gardenia Fruits Extract) (951 mg) was applied to a silica gel column (2.5 cm × 33 cm), and eluted with ethyl acetate:methanol:water (7:1.5:1.5) and then methanol to give 5 fractions (F1, 60 mg; F2, 120 mg; F3, 80 mg; F4, 120 mg; F5, 400 mg). Fraction F2 was separated by preparative HPLC with 80% methanol to give **3** (13.2 mg) and with 60% methanol to give **4** (1.9 mg). Fraction F3 (19.2 mg) was separated by preparative HPLC with 60% methanol to give **2** (10.9 mg) and **5** (1.5 mg). Fraction F4 (85.1 mg) was separated by preparative HPLC with 60% methanol to give **1** (50.0 mg) and **5** (15.3 mg). These compounds were used for the solubility assay.

all-*trans* crocin-1 (**1**): ^1^H-NMR (500 MHz, DMSO-*d*_6_) δ: 7.36 (2H, br d, *J* = 12.0 Hz, 10, 10’), 6.87 (2H, dd, *J* = 2.5, 8.0 Hz, 15, 15’), 6.82 (2H, d, *J* = 15.0 Hz, 12, 12’), 6.67 (2H, dd, *J* = 12.0, 15.0 Hz, 11, 11’), 6.54 (2H, br d, *J* = 8.0 Hz, 14, 14’), 5.42 (2H, d, *J* = 8.0 Hz, 1), 5.32 (2H, br d, *J* = 3.0 Hz, OH), 5.19, 5.10, 4.91, 4.89 (each 2H, br s, OH), 4.85 (2H, br d, *J* = 4.0 Hz, OH), 4.50 (2H, t, *J* = 5.5 Hz, 6’-OH), 4.17 (2H, d, *J* = 8.0 Hz, 1’), 3.99 (2H, br d, *J* = 11.0 Hz, 6), 3.65 (2H, dd, *J* = 5.0, 11.0 Hz 6’), 3.59 (2H, dd, *J* = 5.0, 11.0 Hz 6), 3.40–3.50 (4H, overlapped, 5, 6’), 3.20–3.30 (6H, overlapped, 2, 3, 4), 3.12 (2H, m, 3’), 3.00–3.08 (4H, overlapped, 4’, 5’), 2.95 (2H, m, 2’), 2.00 (6H, s, 20, 20’), 1.97 (6H, s, 19, 19’). ESI-TOFMS: *m/z* 999.3 [M + Na]^+^.

all-*trans* crocin-2 (**2**): ^1^H-NMR (500 MHz, DMSO-*d*_6_) δ: 7.35 (2H, br d, *J* = 11.5 Hz, 10, 10’), 6.87 (2H, dd, *J* = 2.5, 8.0 Hz, 15, 15’), 6.824, 6.817 (each 1H, d, *J* = 14.5 Hz, 12, 12’), 6.67 (2H, dd, *J* = 11.5, 14.5 Hz, 11, 11’), 6.54 (2H, br d, *J* = 8.0 Hz, 14, 14’), 5.421, 5.416 (each 1H, d, *J* = 8.0 Hz, 1, 1’’), 5.33, 5.31, 5.22, 5.14, 5.13, 4.95, 4.92, 4.88, 4.60, 4.47 (each 1H, br s, OH), 4.17 (1H, d, *J* = 8.0 Hz, 1’), 3.99 (1H, br d, *J* = 11.0 Hz, 6), 3.65 (2H, br d, *J* = 11.0 Hz, 6’, 6’’), 3.59 (1H, dd, *J* = 5.0, 11.0 Hz, 6), 3.40–3.45 (4H, overlapped, 5, 5’’, 6’, 6’’), 3.20–3.50 (6H, overlapped, 2, 2’’, 3, 3’’, 4, 4’’), 3.13 (1H, m, 3’), 3.03–3.08 (2H, overlapped, 4’, 5’), 2.95 (1H, t, *J* = 8.0 Hz, 2’), 2.00 (6H, s, 20, 20’), 1.97 (6H, s, 19, 19’). ESI-TOFMS: *m/z* 837.3 [M + Na]^+^.

all-*trans* crocin-3 (**3**): ^1^H-NMR (500 MHz, DMSO-*d*_6_) δ: 7.36 (1H, br d, *J* = 11.5 Hz, 10), 7.21 (1H, br d, *J* = 11.5 Hz, 10’), 6.84–6.87 (2H, 15, 15’), 6.82 (1H, d, *J* = 15.0 Hz, 12), 6.73 (1H, d, *J* = 15.0 Hz, 12’), 6.66 (1H, dd, *J* = 11.5, 15.0 Hz, 11), 6.62 (1H, dd, *J* = 11.5, 15.0 Hz, 11’), 6.49–6.55 (2H, 14, 14’), 5.43 (1H, d, *J* = 8.0 Hz, 1), 5.34, 5.13, 4.92, 4.90, 4.48 (each 1H br s, OH), 4.17 (1H, d, *J* = 8.0 Hz, 1’), 3.99 (1H, br d, *J* = 11.0 Hz, 6), 3.65 (1H, br d, *J* = 11.0 Hz, 6’), 3.59 (1H, dd, *J* = 5.0, 11.0 Hz, 6), 3.40–3.50 (2H, overlapped, 5, 6’), 3.22–3.28 (3H, overlapped, 2, 3, 4), 3.13 (1H, t, *J* = 8.5 Hz, 3’), 3.05–3.08 (2H, overlapped, 4’, 5’), 2.96 (1H, t, *J* = 8.0 Hz, 2’), 1.989, 1.986 (each 3H, s, 20, 20’), 1.97 (3H, s, 19), 1.92 (3H, s, 19’). ESI-TOFMS: *m/z* 675.3 [M + Na]^+^.

all-*trans* crocin-4 (**4**): ^1^H-NMR (500 MHz, DMSO-*d*_6_) δ: 7.35 (2H, br d, *J* = 11.5 Hz, 10, 10’), 6.87 (2H, dd, *J* = 2.5, 8.0 Hz, 15, 15’), 6.82 (2H, d, *J* = 15.0 Hz, 12, 12’), 6.67 (2H, dd, *J* = 11.5, 15.0 Hz, 11, 11’), 6.54 (2H, br d, *J* = 8.0 Hz, 14, 14’), 5.42 (2H, d, *J* = 8.0 Hz, 1’’), 5.31, 5.15, 5.04, 4.59 (each 2H, br s, OH), 3.65 (2H, br d, *J* = 10.5 Hz, 6’’), 3.45 (2H, m, 6’’), 3.12–3.25 (6H, overlapped, 3’’, 4’’, 5’’), 3.14 (2H, t, *J* = 9.5 Hz, 2’’), 2.00 (6H, s, 20, 20’), 1.97 (6H, s, 19, 19’). ESI-TOFMS: *m/z* 675.3 [M + Na]^+^.

13-*cis* crocin-1 (**5**): ^1^H-NMR (500 MHz, DMSO-*d*_6_) δ: 7.48 (1H, br d, *J* = 15.0 Hz, 10), 7.46 (1H, br d, *J* = 12.0 Hz, 10’), 7.37 (1H, br d, *J* = 12.0 Hz, 12), 7.18 (1H, br dd, *J* = 11.5, 14.5 Hz, 12’), 6.82 (1H, d, *J* = 15.0 Hz, 12’), 6.77 (1H, br dd, *J* = 11.5, 14.5 Hz, 15’), 6.65 (2H, dd, *J* = 12.0, 15.0 Hz, 11, 11’), 6.49 (1H, br d, *J* = 11.5 Hz, 14’), 6.40 (1H, br d, *J* = 11.5 Hz, 14), 5.45, 5.43 (each 1H, d, *J* = 8.0 Hz, 1), 5.32, 5.19, 5.10, 4.92, 4.89, 4.86, 4.45 (each 2H, br s, OH), 4.174, 4.171 (each 1H, d, *J* = 8.0 Hz, 1’), 4.00, 3.99 (each 1H, br d, *J* = 10.0 Hz, 6), 3.65 (2H, br dd, *J* = 3.5, 11.0 Hz, 6’), 3.59 (2H, br dd, *J* = 5.0, 11.0 Hz, 6), 3.41–3.45 (4H, overlapped, 5, 6’), 3.21–3.30 (6H, overlapped, 2, 3, 4), 3.12 (2H, m, 3’), 3.03–3.08 (4H, overlapped, 4’, 5’), 2.96 (2H, br t, *J* = 8.0, 2’), 2.00 (3H, br s, 20), 1.98 (3H, br s, 20’), 1.98 (3H, br s, 19), 1.97 (3H, br s, 19’). ESI-TOFMS: *m/z* 999.5 [M + Na]^+^.

### HPLC based solubility assay

Berberine hydrochloride was dissolved in water at 60 ºC to prepare 2 mM solution. Baicalin was dissolved in methanol to prepare 4 mM solution. The assay samples were dissolved in dimethyl sulfoxide (DMSO) and diluted 100 times with water to adjust their concentration at 800 µg/mL (1% DMSO). Solutions of berberine hydrochloride (200 µL), baicalin (100 µL), and a sample (500 µL) were added to a 1.5 mL-Eppendorf tube in this order, mixed for 30 s with VORTEX-GENIE 2 Mixer (M&S Instruments Inc., Osaka, Japan), and left for 60 min at room temperature. The mixture was centrifuged at 13,000*g* for 10 min, and then 100 µL of the supernatant was concentrated to dryness under reduced pressure. The residue was dissolved in 100 µL of methanol and analyzed by HPLC. One percent of DMSO was used as a control solution.

As a measure of solubility-enhancement activity, relative solubilities of berberine and baicalin in an assay mixture compared to the control solution (without any sample) were calculated from their peak areas of HPLC chromatogram monitored at UV 275 nm by the following equation:

Relative solubility (%) = $$\frac{{\text{peak area of berberine or baicalin in an assay}}}{{\text{peak area of berberine or baicalin in the control (DMSO)}}}$$ × 100 (%).

### Statistical analysis

The significant differences in the relative solubilities between the control and the assay samples were tested by Dunnett’s multiple comparison test using *R* statistical software. The results of the relative solubility of berberine and baicalin were analyzed statistically by a two-tailed Student’s *t* test.

## Results

### Identification of the constituents with solubility-enhancement activity from Gardenia Fruit

**Fig. 3:**
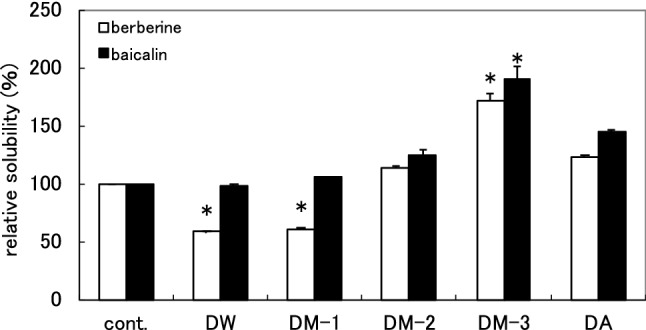
Solubility-enhancement of berberine–baicalin complex by DW, DM-1, DM-2, DM-3, and DA at 500 µg/mL The results are expressed as the average of two independent experiments. An asterisk indicates a significant difference with control (*p* < 0.01). cont.: without sample

To isolate compounds that enhance the solubility of berberine–baicalin complex from Gardenia Fruit, activity-guided fractionation was conducted. Gardenia Fruit was extracted with hot water, then the extract was fractionated by a Diaion HP-20 column eluted with water (DW), 20% MeOH (DM-1), 50% MeOH (DM-2), MeOH (DM-3) and acetone (DA). The solubility-enhancement activity of each fraction was evaluated by HPLC based solubility assay (Fig. 3). Fraction DM-3 showed significant solubility-enhancement (the relative solubilities of berberine and baicalin at 500 µg/mL were 172% and 191%, respectively). Similarly, DM-2 and DA also showed the activity though the potency was lower than in the DM-3. In TLC analyses of these fractions, yellow spots were detected in DM-2, DM-3, and DA, whereas no yellow spot was observed in both DW and DM-1. These yellow spots corresponded to those detected in commercially available crocin (Gardenia Fruit extract). These results suggested that solubility-enhancement activity observed in DM-2, DM-3 and DA was attributable to crocins.

**Fig. 4:**
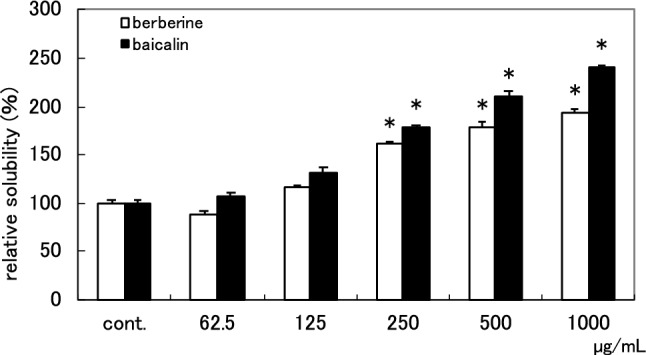
Solubility-enhancement of berberine–baicalin complex by commercially available crocin at different concentrations The results are expressed as the mean ± SD of three independent experiments. An asterisk indicates a significant difference with control (*p* < 0.01). cont.: without sample

Next, to evaluate the solubility-enhancement activity of crocins, the activity of commercially available crocin was measured at various concentrations (Fig. 4). It showed concentration-dependent activity, and the relative solubilities of berberine and baicalin at 500 µg/mL were 179% and 211%, respectively. These results indicated that crocins contained in Gardenia Fruit were responsible for the solubility-enhancement activity to the baicalin–berberine complex.

### Isolation of crocins (1–5)

**Fig. 5:**
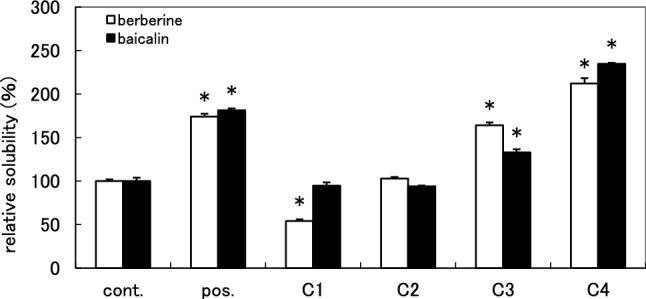
Solubility-enhancement of berberine–baicalin complex by fractions C1–C4 at 500 µg/mL The results are expressed as the mean ± SD of three independent experiments. An asterisk indicates a significant difference with control (*p* < 0.01). cont.: without sample, pos.: commercially available crocin (500 µg/mL)

For the isolation of crocins, Gardenia Fruit was extracted with 40% aqueous ethanol, and then the extract was concentrated and partitioned between water and ethyl acetate [14]. The water layer was applied to a Diaion HP-20 column, eluted with water–methanol (0%, 20%, 50%, and 100% methanol) to give four fractions (C1, C2, C3, and C4, respectively). Fraction C4 mainly contained crocins (by TLC analysis) and showed significant activity (Fig. 5). Fraction C4 was repeatedly separated by preparative HPLC to give compounds **1**–**4** (Fig. 1). To obtain crocins more effectively, commercially available crocin was fractionated by silica gel column chromatography and separated by preparative HPLC to give compounds **1**–**5**. Compounds **1**–**3** [17], **4** [18] and **5** [19, 20] were identified by comparisons of their spectral data with those reported in the literature.

### Solubility-enhancement activities of crocins and their mixture

**Fig. 6:**
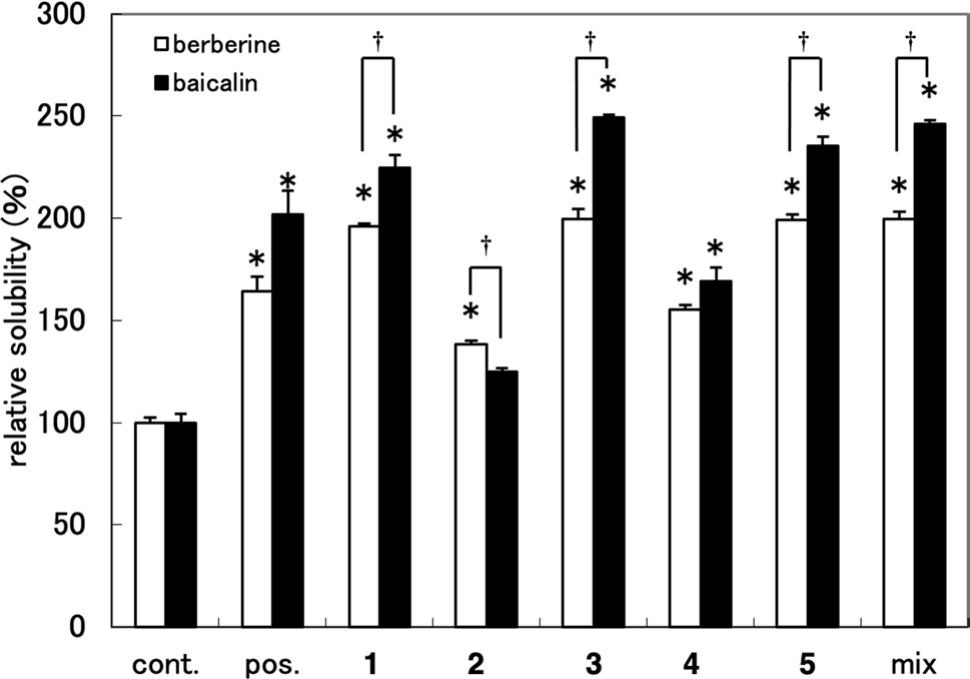
Solubility-enhancement of berberine–baicalin complex by compounds **1**–**5** and their mixture at 500 µg/mL The results are expressed as the mean ± SD of three independent experiments. An asterisk indicates a significant difference with control (*p* < 0.01). A dagger indicates a significant difference between baicalin and berberine (*p* < 0.05). cont.: without sample, pos.: commercially available crocin (500 µg/mL)

To evaluate the solubility-enhancement activity of isolated crocins **1**–**5**, their relative solubilities for berberine and baicalin in the presence of **1**–**5** alone and also of their mixture were measured (Fig. 6). Three compounds (**1**, **3**, and **5**) and the mixture, whose ratio of **1**–**5** was identical to that found in the commercially available crocin, showed high activity for berberine (about 200%) and baicalin (about 235%), whereas the activities of compounds **2** and **4** were lower. Compounds **1**, **3** and **5** showed the same level of activity at 500 µg/mL. However, as the molecular weights of **1** and **5** (MW 976) are larger than that of **3** (MW 652), the activities of **1** and **5** in molar concentration basis were higher than that of **3**.

The mixture of **1**–**5** showed the same level of solubility-enhancement with **1** and **5**. This result suggested that these compounds act in the same mechanism for solubility-enhancement. The commercially available crocin (positive control), whose HPLC chromatogram implied the presence of compounds other than crocins (Fig. 2), showed lower activity than the crocin mixture (Fig. 6). The relative solubilities of commercially available crocin were 164% and 202%, and those of the crocin mixture were 200% and 246%, for berberine and baicalin, respectively. As the total content of crocins in the commercially available crocin was about 80% expected from the HPLC chromatogram (Fig. 2b), these results seemed well corresponded. Compounds **1**, **3**, **5** and the crocin mixture showed significantly higher activity to baicalin than berberine, indicating that solubility of baicalin was more enhanced by these crocins than that of berberine.

## Discussion

Orengedokuto has been a representative prescription for heat-clearing and detoxicating and used to treat inflammatory diseases [21]. In a previous paper, we showed that berberine, the constituent of Coptis Rhizome and Phellodendron Bark, contributed to the anti-inflammatory activity of orengedokuto through inhibition of nitric oxide production [7]. We also revealed that flavonoids of Scutellaria Root such as baicalein and wogonin played a central role in the inhibition of prostaglandin production by orengedokuto [22, 23]. These flavonoids are mainly contained as glycosides in Scutellaria Root and absorbed as themselves and as their aglycon [24]. Berberine forms a complex with baicalin and wogonoside, the major flavone glycosides of Scutellaria Root whose aglycon are baicalein and wogonin, respectively, and precipitates out from aqueous solution [4, 6]. This means that the anti-inflammatory constituents of orengedokuto would be reduced in the process of decoction, leading to be less effectiveness. In fact, a large amount of precipitates, which mainly consisted of berberine and baicalin, was observed in a decoction prepared from Coptis Rhizome, Phellodendron Bark and Scutellaria Root. However, the amount of the precipitates was largely decreased in a decoction of orengedokuto, which contained Gardenia Fruit in addition to the mixture [7]. In this study, through activity-guided fractionation, we identified crocins as the compounds in Gardenia Fruit involved in the solubility-enhancement of berberine–baicalin complex. Thus, Gardenia Fruit contributes anti-inflammatory activity of orengedokuto by increasing solubilities of anti-inflammatory constituents of the other component crude drugs in the prescription. In our study, we focused on anti-inflammatory activity of orengedokuto. However, other pharmacological activities of berberine and baicalin have been reported. Berberine inhibits α-glucosidase [25], exhibits antidiarrheal effect [26], and has antibacterial activity [27], and baicalin shows antithrombotic activity [28] etc. Therefore, Gardenia Fruit may also be involved in other therapeutic effects of orengedokuto. Thus, our result will add scientific basis to the understanding of the effectiveness of orengedokuto as a whole.

Kampo prescriptions and their effectiveness have been established through many experiences of applications to patients. However, the scientific basis of the effectiveness of Kampo prescriptions, especially how interactions among constituents contribute to the effect of a Kampo prescription, is still limited. Further investigations based on the constituents to understand the effectiveness of a Kampo prescriptions will be necessary.

Berberine and baicalin are reported to form a 1:1 complex [4, 6], and Wang et al*.* reported that carboxyl group of baicalin and the quaternary ammonium ion of berberine are facing inside, and hydrophobic parts of baicalin and berberine are outside in the complex [6]. As the crocins with solubility-enhancement activity have a large hydrophobic polyene moiety and hydrophilic glycoside parts on both sides, and the activity seemed correlated with the number of sugars, they may act as a surfactant to dissolve the complex. However, as the relative solubilities of berberine and baicalin are different (Fig. 6), crocins may act differently to berberine and baicalin. Investigations on the mechanism of the solubility-enhancement of crocins are in progress in our laboratory and will be discussed in a forthcoming report. In the highly polar fractions [DW and DM-1 (Fig. 3), C1 (Fig. 5)], the solubility of berberine was lowered while that of baicalin was the same level with the control. This suggested the presence of compounds which specifically interact with berberine in these fractions. Further investigation will be necessary to identify the compounds.
